# The Effect of Processing Variables on Powder Interlayer Bonding in Nickel-Based Superalloys

**DOI:** 10.3390/ma13030601

**Published:** 2020-01-29

**Authors:** Olivia Stanners, Sean John, Helen M. Davies, Ieuan Watkins, Silvia Marchisio

**Affiliations:** 1 Institute of Structural Materials, Bay Campus, Swansea University, Swansea SA1 8EN, UK; s.e.john@swansea.ac.uk (S.J.); h.m.davies@swansea.ac.uk (H.M.D.); 919341@swansea.ac.uk (I.W.); 2 Rolls-Royce plc, P.O. Box 31, Derby DE24 8BJ, UK; Silvia.Marchisio2@Rolls-Royce.com

**Keywords:** powder, interlayer, bonding, joining, nickel-based superalloys

## Abstract

Powder Interlayer Bonding (PIB) has been considered as a lower-energy joining technology for nickel-based superalloys compared to conventional methods; such as friction welding. Typically; nickel-based superalloys exhibit high energy requirements for joining due to their high operating temperatures. However; PIB utilizes a localized temperature gradient created by an induction current; reducing the energy requirements for the process. PIB is a solid-state joining method that compresses and heats a powder interlayer between two faying surfaces to produce one joined workpiece. It has been successfully used to bond titanium alloys; and the objectives of this work were to explore its application as a joining method for nickel-based superalloys. Initial results showed that joining nickel-based superalloys via PIB is possible; and bondlines with very little porosity were observed. Further analysis showed that these bonded areas had lower porosity than the base material; suggesting PIB could be a successful joining method for difficult-to-join nickel-based superalloys.

## 1. Introduction

Nickel-based superalloys are favoured for use in the combustor and turbine sections of gas turbine engines due to their superior performance at high temperatures. Advances have been made in their design, such that they can withstand average temperatures of 1050 °C, with the alloys offering fatigue, creep resistance, and resistance to corrosion even at the most elevated service temperatures experienced in the compressor and turbine section of the engines [1].

Nickel-based superalloys often contain 10%–20% chromium, along with small additions of over 10 other alloying elements that play a key role in the microstructure of the overall alloy. The matrix of these alloys is a continuous face centered cubic (FCC) austenitic phase, known as the γ phase. This phase is rich in alloying elements such as cobalt, chromium, and molybdenum that act to strengthen the alloy. Contained within the FCC γ phase is the precipitate phase of γ’; additions of aluminum and titanium cause γ‘ to coherently precipitate within the FCC γ matrix. Carbides are also present within the microstructure due to small carbon additions within the alloy of around 0.05%–0.2%. These additions react with other elements present within the composition to form MC carbides (such as M_22_C_6_, where M is a metallic atom such as Ti, Hf and Ta) [2].

Novel joining techniques are of high importance in the aerospace industry, as 8% of operating costs for airline operators are for engine maintenance and repair [3]. Powder Interlayer Bonding (PIB) is of interest as a potential technique for the joining and repair of aerospace alloys. It has successfully been used as a joining technique for titanium alloys (Ti-64 and TI-6246) [4,5], and is here explored as a potential technology to join nickel-based superalloys.

PIB is a solid-state joining method that has aspects similar to both diffusion bonding and diffusion brazing. Diffusion brazing creates material coalescence through heating the workpieces and using a filler metal between the two faying workpieces, that are to be joined. The filler metal used in brazing has a liquidus temperature below that of the parent metals, and this allows for the filler metal to be in liquid state for joining while parent metals remain in a solid state at the elevated temperature of the brazing process. The amount of time allowed for diffusion brazing needs to be adequate to allow the filler material to interact with the solid parent material, so it can diffuse to alter the joint properties to approach the properties of the parent materials [6].

Diffusion bonding involves the joining of two faying surfaces via exposure to an elevated temperature for an extended time period. During time at elevated temperatures, the materials being joined diffuse into one another, joining them to create one workpiece [7,8]. Unlike diffusion brazing, diffusion bonding is solid-state, does not involve the use of an interlayer, and all materials remain in the solid state throughout the bonding process.

PIB is a joining process that exploits a localized temperature gradient and uniaxial pressure within a stream of inert gas and or vacuum. It involves the use of a powder interlayer between the two faying surfaces that are to be joined, similar to how diffusion brazing uses a filler metal. The two specimens are then joined through application of compressive force and heat. Compressive force aids in the diffusion of the power interlayer into the two separate specimens to be joined, and helps reduce the effect of surface asperities. Surface asperities are an unavoidable feature of any surface, even ones that seem to be free of scratches; they are rough features that expand out of the material surface, and when bonding occurs without an external force being applied, the only contact between the two surfaces being bonded is these asperities. When an external force is applied, such as in PIB, these asperities plastically deform, and the area of contact between the two surfaces is increased aiding diffusion across the workpiece by increasing the available areas for diffusion to occur.

Although heating in PIB is localized to the area of the specimen with the powder applied, a heat-affected zone (HAZ) is created, as areas of the specimen close to the induction current that creates the heat also unavoidably increase in temperature. The combination of compressive force, elevated temperature and the time for which the specimens are exposed to these conditions create the powder interlayer bond between the two specimens creating one joined workpiece. The inert atmosphere in which bonding is performed reduces oxidation during the process [4].

## 2. Materials and Methods

As machined, 10 mm diameter specimens of a new generation nickel-based superalloy were supplied by Rolls–Royce plc. Gas atomized powder of the same composition was supplied for use in the interlayers. The as-received powder was subsequently sieved to a size range of sub-32 µm. The interlayer consisted of powder, deionized water, cellulose, and glycerol to form a paste. The paste was applied to one specimen surface using a specially manufactured jig that allowed 150 µm layers to be accurately measured onto the surface.

**Figure 1:**
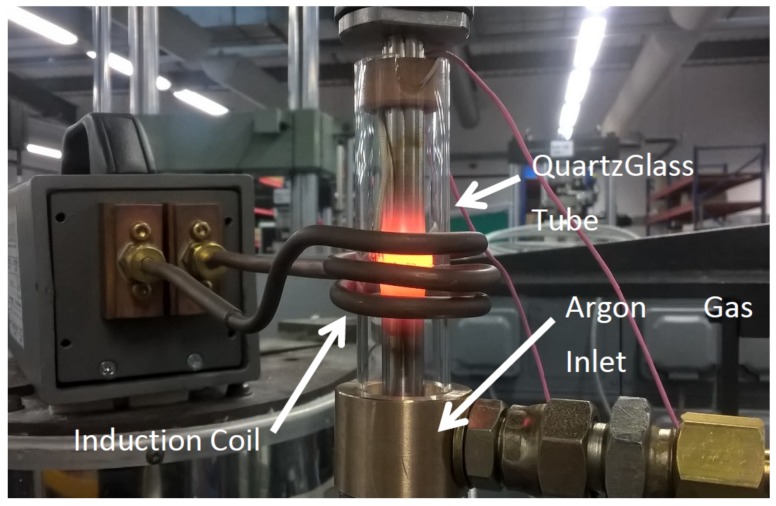
Powder Interlayer Bonding (PIB) setup.

The rig setup used for the PIB process is shown in Figure 1. The two specimens, to be joined, were secured in a servohydraulic rig via collets, and their faying surfaces were brought together under very low force (˂0.5 kN), so that they were just touching. The specimens were then subjected to elevated temperatures within a 1000–1100 °C temperature range, using a water-cooled induction coil positioned to create a localized temperature gradient around the powder interlayer. Once the interlayer region was at the desired temperature, a force of 5–10 kN was applied. Bond temperature was measured using N-type thermocouples connected to a calibrated Fluke 54 II thermometer. The thermocouples were spot-welded onto the specimen within 1 mm of the faying surfaces. The specimens were held under load and at temperature for 5–10 min. Certain tests were held at the elevated temperature for an additional 30 min, but with the force reduced to 0.5 kN. The parameters for each bond completed are shown in Table 1. The bonding procedure was performed in an argon environment to prevent oxidation.

Once the specimens were successfully bonded as one workpiece, percentage deformation was measured at the interlayer by calculating the change in diameter.

(1) $$Deformation\;\left(\%\right)=\left(\frac{Change\;in\;diameter}{Original\;diamter}\right)\times 100$$

For microstructural analysis, sections were taken around the interlayer region and mounted in conductive Bakelite, prior to preparation by standard grinding and polishing sequences.

A Hitachi SU3500 scanning electron microscope (Hitachi, Tokyo, Japan) was used to capture Scanning Electronic Microscopy (SEM) images of the bondlines and the same microscope. Electron Backscatter Diffraction (EBSD) data were captured via an Oxford Instruments EBSD camera (Oxford, UK) and subsequently analyzed with Channel 5 Tango software attached to the Hitachi SU3500. The specimens were not etched for SEM or EBSD analysis.

All field emission gun scanning electron microscopy (FEG-SEM) images were captured using a JEOL 7800 FEG-SEM (JEOL, Tokyo, Japan). The prepared specimens were electroetched using a 10% phosphoric acid solution prior to FEG-SEM analysis.

An image from the centre of each bondline was captured on the SEM at the same magnification for each bond, and then analyzed using ImageJ software (1.51k, National Institute of Health, Bethesda, MD, USA) to obtain porosity measurements. On the ImageJ software, the “threshold” function was selected and set to the same threshold value for each bondline, so pores within the bondline and surrounding base material appeared red. The software then calculated the number and area of the pores present.

**Figure 2:**
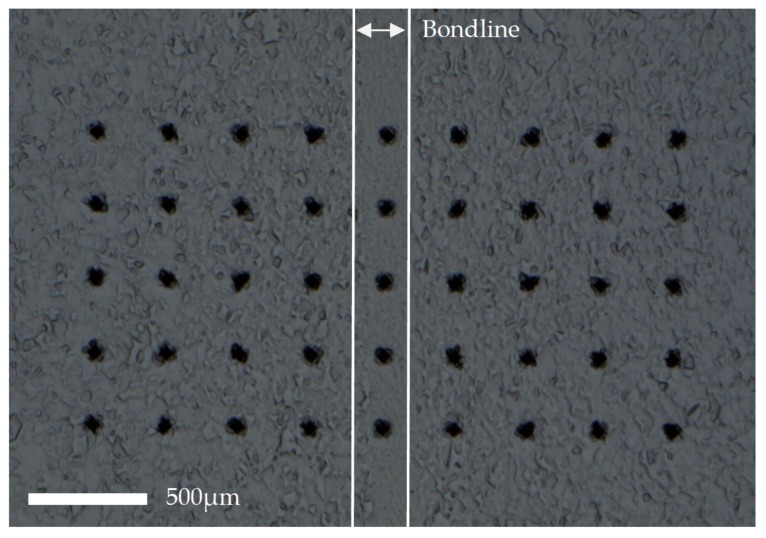
Constructed 9 × 5 grid of Vickers hardness indents.

Vickers hardness measurements were conducted on a Struers Duramin-40 A3 machine with a 9 × 5 grid constructed using 1 kgf, spacing of 0.3 mm, and 10 s dwell time (shown in Figure 2). The 5th point on each row coincided with the bondline on every specimen.

## 3. Results and Discussion

### 3.1. Microstructure of Base Material (As-Received)

**Figure 3:**
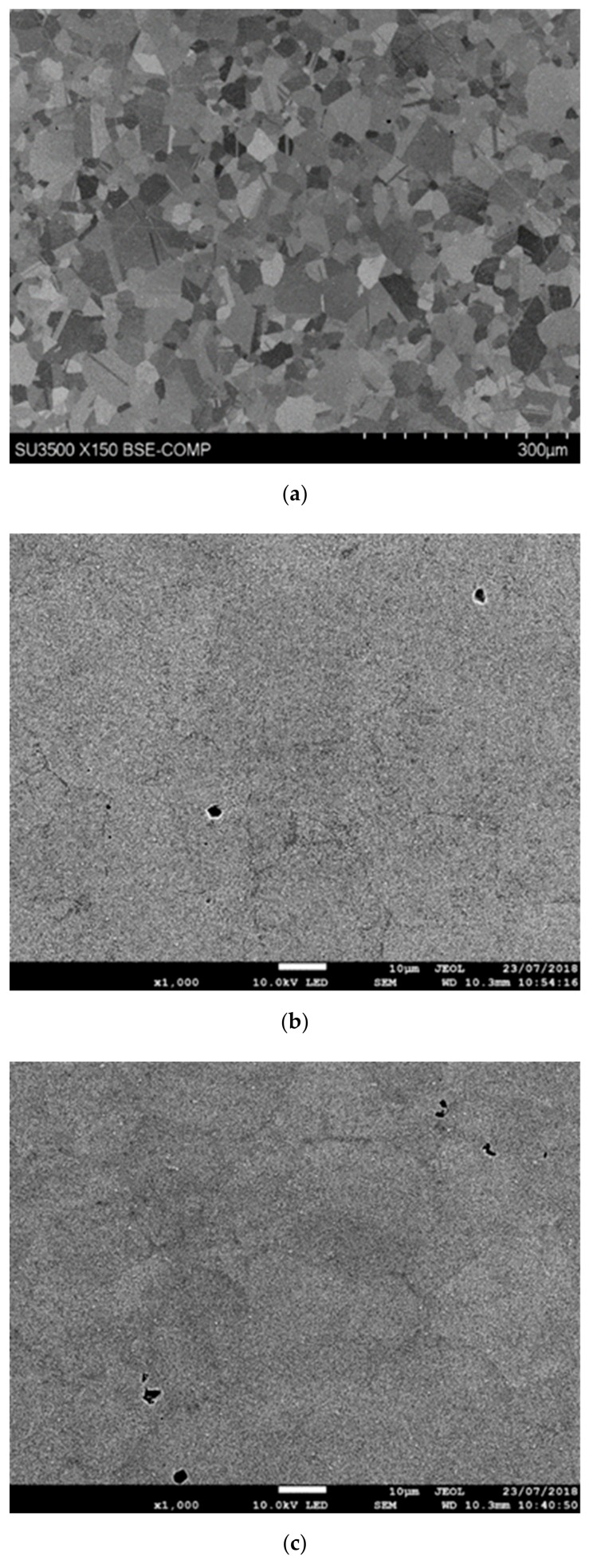
(**a**) Scanning Electron Microscope (SEM) images of as-received base material (**b**) and (**c**) FEG SEM images of as-received base material.

**Figure 4:**
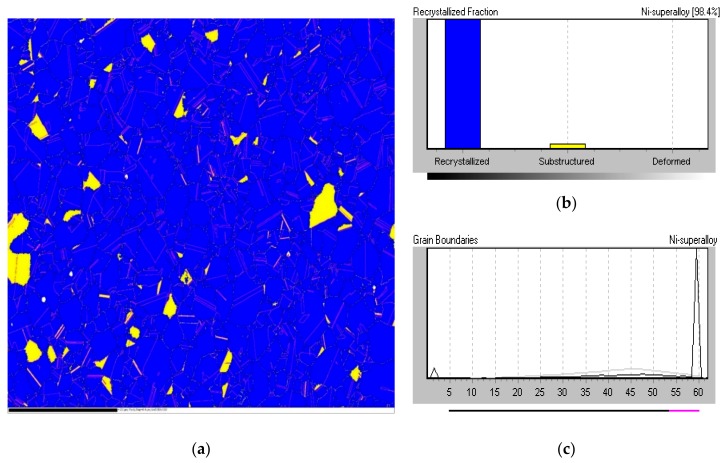
Electron backscatter diffraction (EBSD) data for, (**a**) image of recrystallization fraction of, (**b**) graph of recrystallization fraction of, and (**c**) grain boundaries within as-received base material.

Figure 3a–c shows the microstructure of the nickel-based superalloy in the as-received condition before any bonding took place; prior to elevated temperature exposure. Clearly defined grains and twin boundaries are identifiable in Figure 3a. Figure 3b,c show that pores were present throughout the material. Grain-size data obtained from EBSD analyses are shown in Table 2 and indicate a range of grain sizes, varying from 5.5 to 76 µm. Figure 4a,b show the material to comprise mainly recrystallized grains, with very few substructured grains and the absence of any deformed grains. Figure 4c shows the grain boundaries present within the microstructure; the peak observable at 60° indicates the existence of a large-volume of twin crystals.

### 3.2. Microstructure within Heat-Affected Zone

**Figure 5:**
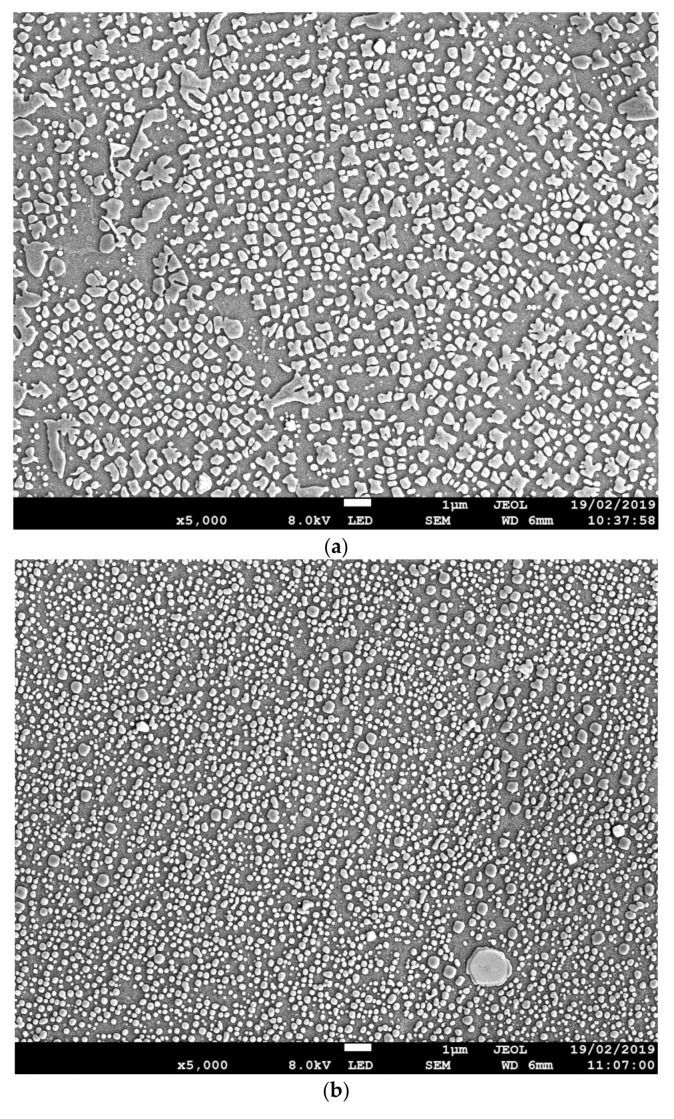

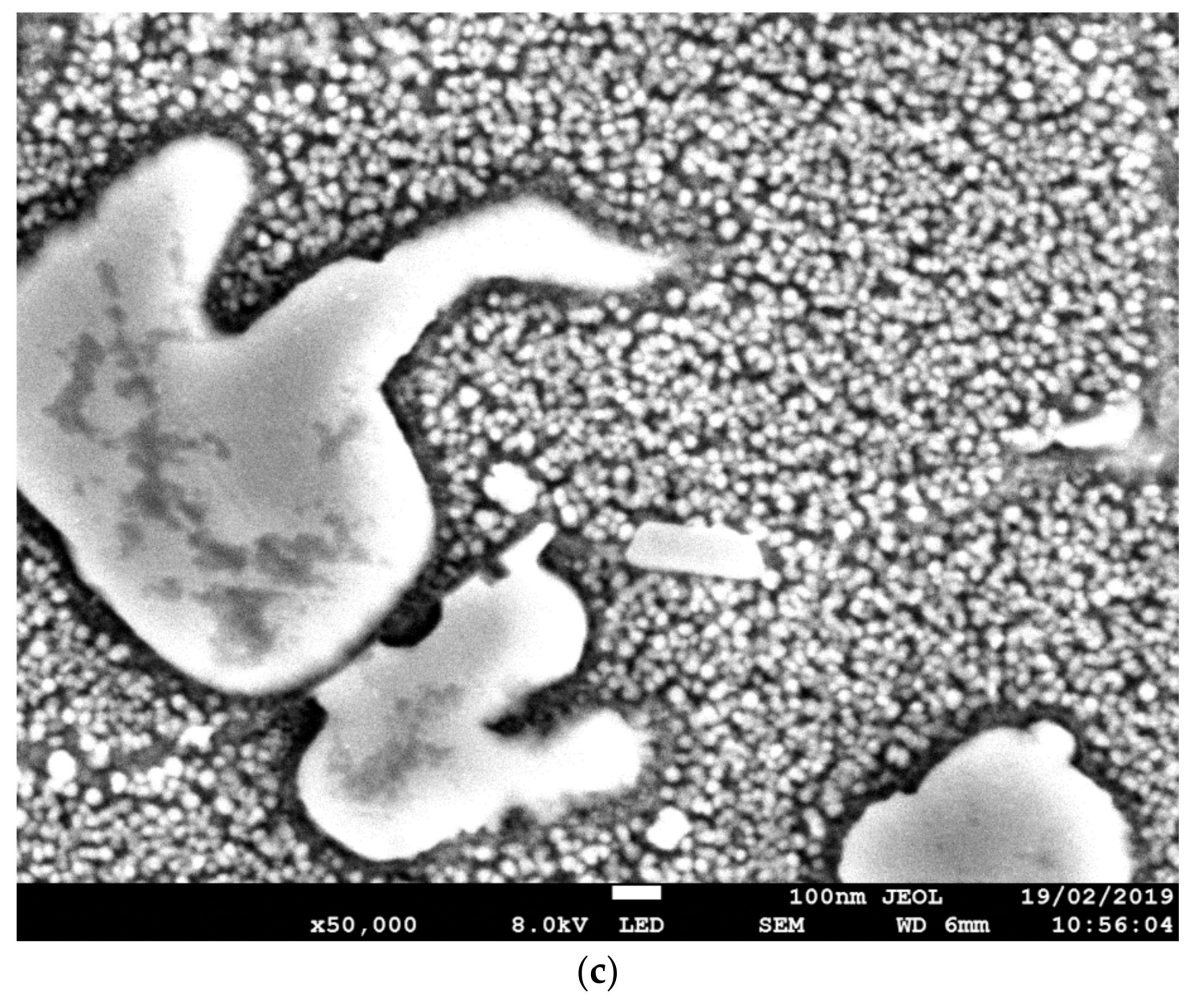
(**a**) FEG SEM image approximately 3 mm from the bondline, (**b**) FEG SEM image approximately 1 mm from the bondline, and (**c**) FEG SEM image approximately 1 mm from the bondline.

Figure 5a–c show the microstructural variations throughout the HAZ as the bondline is approached. The γ background matrix was the same throughout the HAZ, but the size and shape of secondary γ’ precipitates changed as the bondline was approached. Temperatures at the bondline, where the localized induction heating was positioned, were the highest. Areas surrounding the bondline had unavoidably been heated, leading to the formation of the observable HAZ. Tertiary γ’ dissolved into the γ matrix at approximately 900 °C, and primary γ’ was not present due to the processing of the material prior to bonding. Therefore, the main precipitate within the microstructure was secondary γ’. Near the bondline (Figure 5b), the secondary γ’ was small and spherical in shape compared to areas further away (Figure 5a), where it was more cuboidal and larger in size. At the highest magnifications, it was possible to observe the tertiary γ’ located within the γ matrix that reprecipitated out from the γ solution as the material cooled (Figure 5c).

### 3.3. Bondline Microstructure 

**Figure 6:**
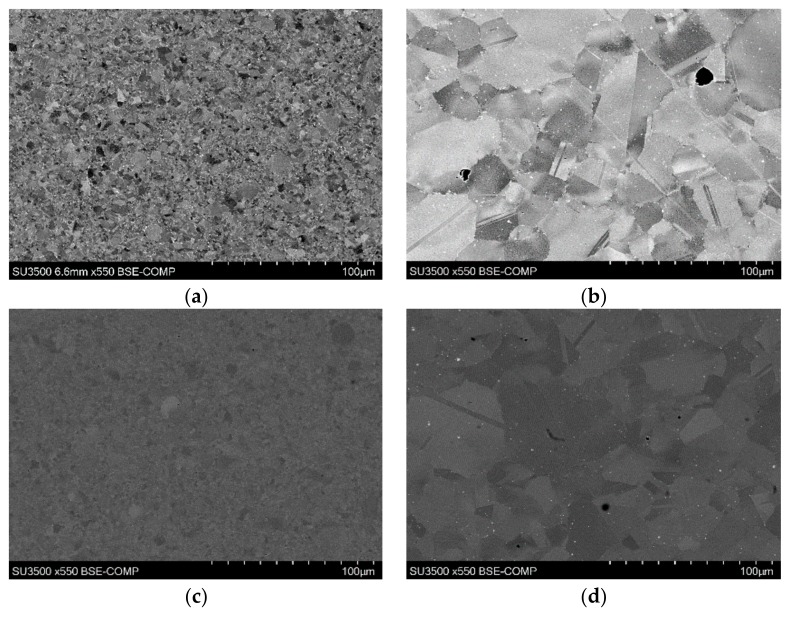

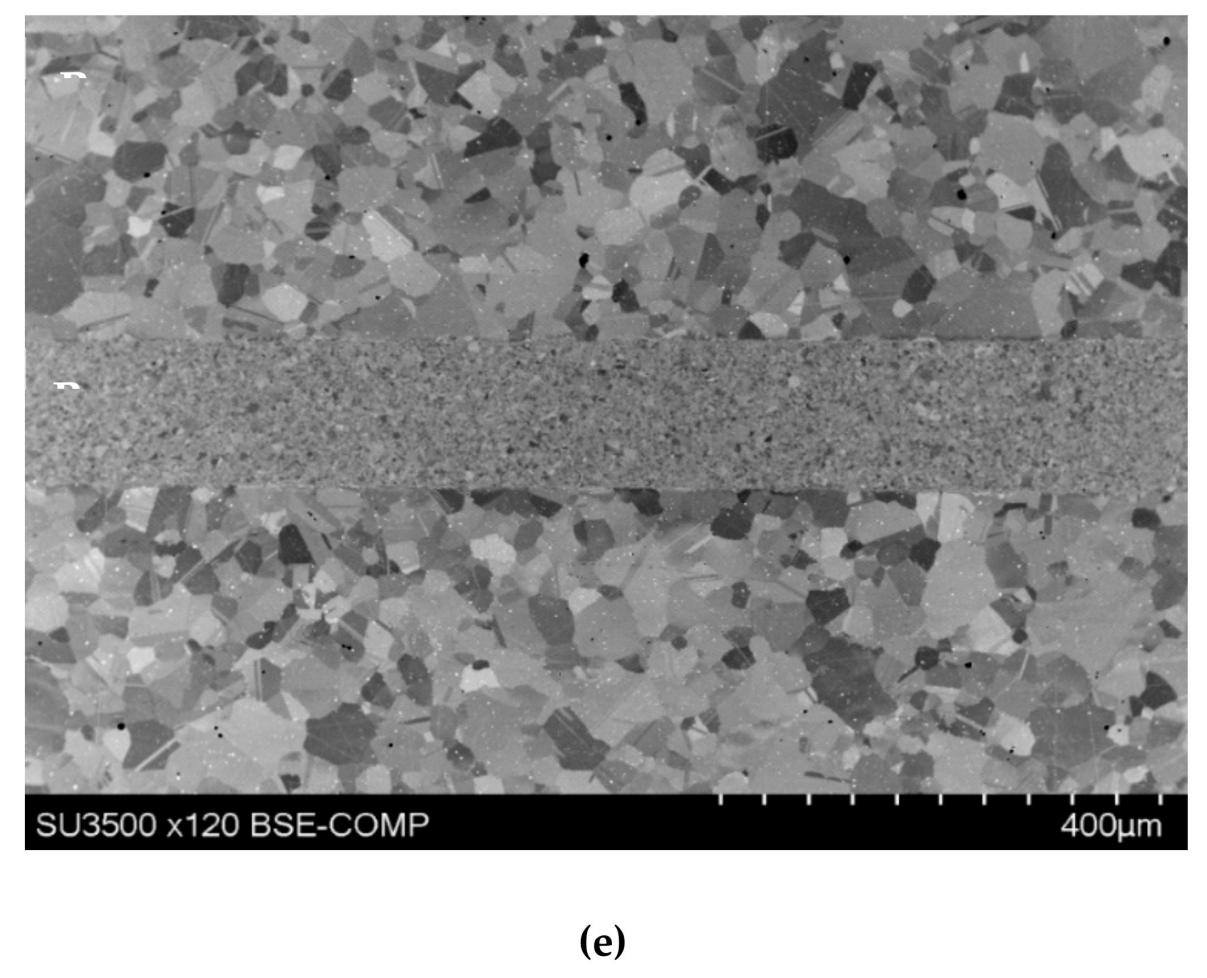
SEM images of (**a**) bondline 9, (**b**) bond 9 base material, (**c**) bondline 12, (**d**) bond 12 base material, and (**e**) bond 4 showing variation in grains from bondline to base material.

The bondline region had a finer microstructure than that of the surrounding base material. The surrounding microstructure was comparable to that of the as-received base material, whereas the much finer microstructure observed at the bondline appeared to show that a transformation occurred where the material was subjected to the most elevated temperatures as shown in Figure 6a–e.

**Figure 7:**
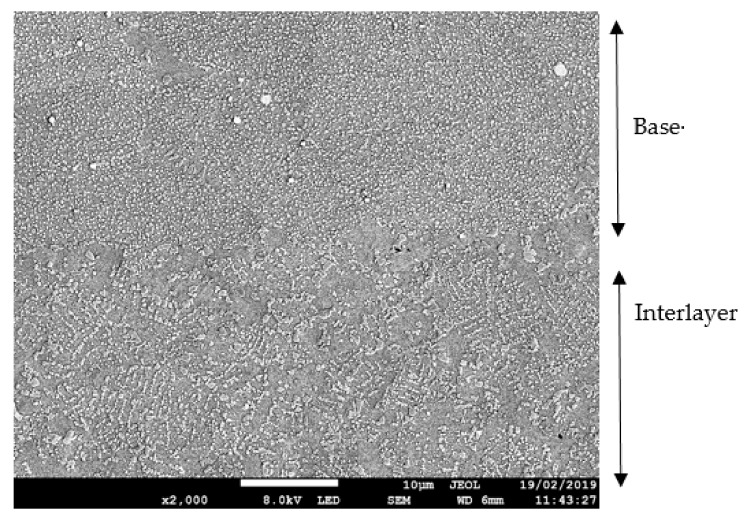
FEG SEM image of interlayer in bond 1.

Figure 7 shows that the secondary γ‘ was more sparsely distributed within the interlayer compared to the surrounding base material. The reduced presence of this strengthening precipitate within the interlayer is not desirable when the mechanical properties of the material are considered, as a reduction in strength at the interlayer results in a weakened area where fractures and failures can occur.

### 3.4. Microstructure with Varying Parameters

**Figure 8:**
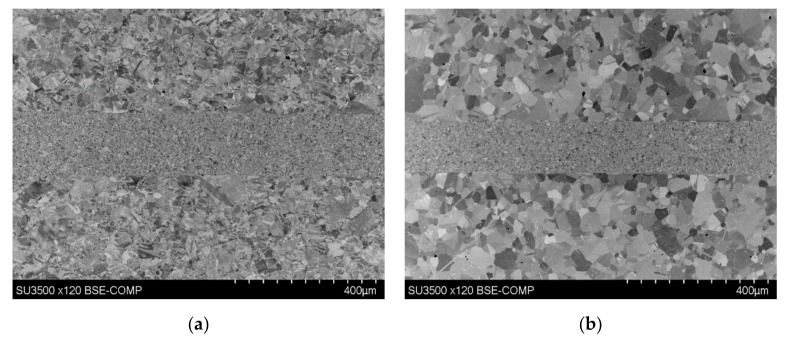
SEM images of (**a**) bond 3 (bonded at 1100 °C) and (**b**) bond 4 (bonded at 1050 °C).

Temperature has been shown to affect the base material microstructure. At higher bonding temperatures the base material, surrounding the bondline, appeared to have a finer structure compared to the base material when bonded at lower bonding temperatures, as shown in Figure 8. The increased temperature of bonding increased the driving energy for recrystallization and in lower temperature bonding where recrystallization had not occurred in the material surrounding the bondline insufficient energy must have been present due to the lowered temperatures. In specimens that had increased amounts of upset, the strain this upset induced acted to drive recrystallization [9]. Bond 4 experienced a higher bonding temperature than bond 3 (1100 °C vs. 1050 °C) and this resulted in a higher degree of deformation (3% in bond 4 vs. 0.26% in bond 3) of the bonded specimen. This combination of increased temperature and strain energy proved sufficient to drive the recrystallization process. Increased bonding time for bond 3 would have allowed for grain growth to occur resulting in an increased average grain size for the recrystallized material surrounding the bondline [10].

### 3.5. Hardness of Bond Region

**Figure 9:**
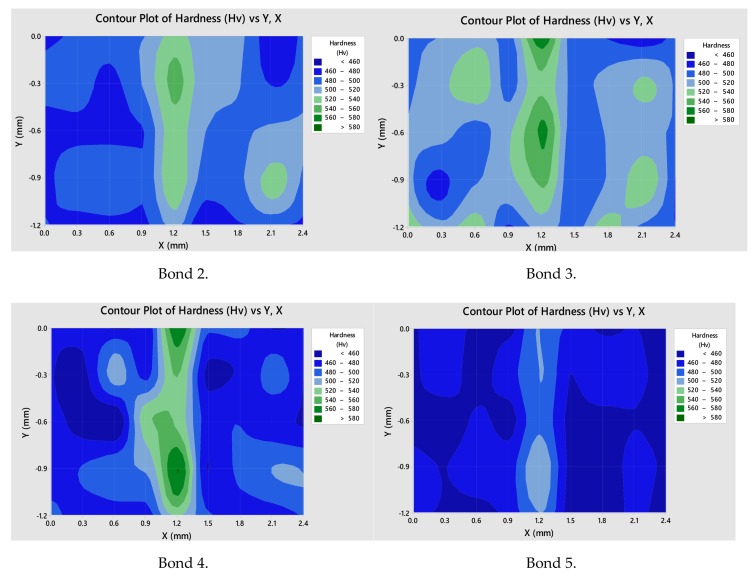

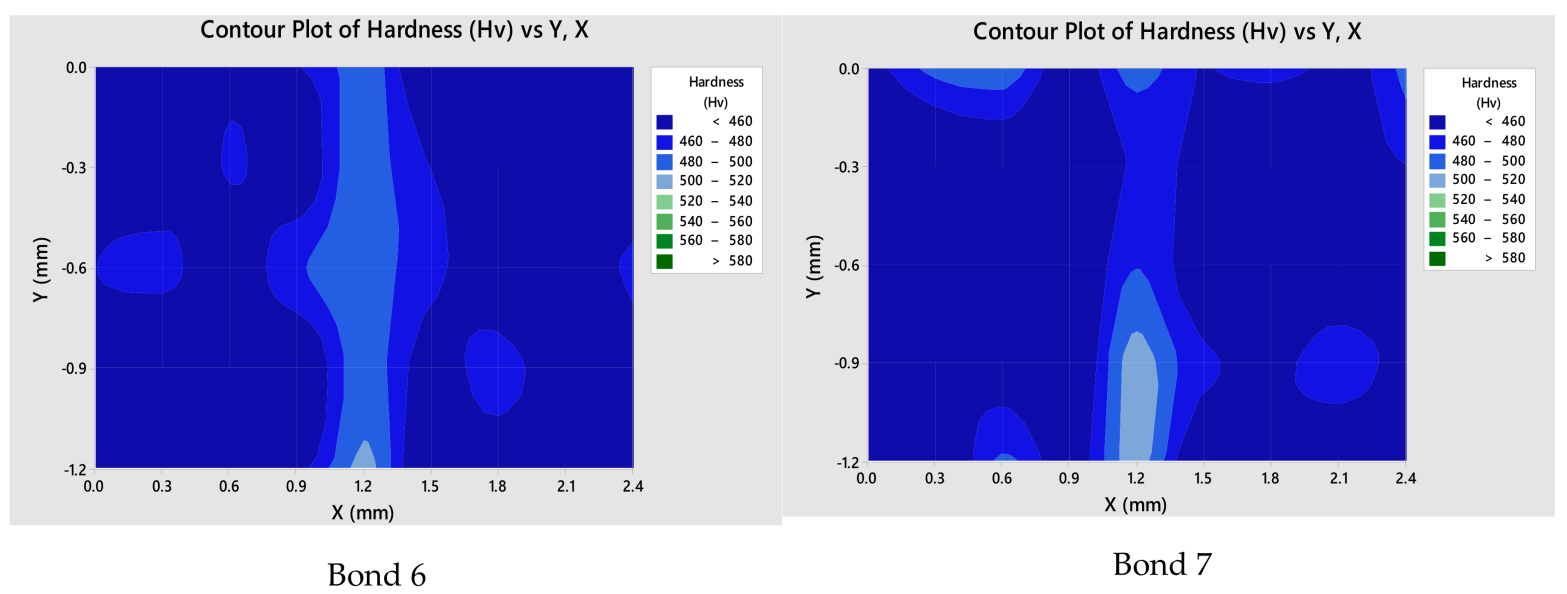
Contour maps showing variation in hardness for bondlines.

In Figure 9, the bondline is represented at X = 1.2 mm, the hardness values within the bondline were higher than those measured in the surrounding material. The area immediately surrounding the bondline also had higher hardness values than the material located further away from the bondline. This material was located within the HAZ and would have therefore undergone some microstructural changes within the PIB process, with secondary γ’ becoming more spherical with a finer structure, but more frequent distribution within the microstructure, as shown in Figure 5.

The harder material located at the bondline corresponded to the finer microstructure observed in Figure 6a,c,e. This finer-grain material was harder than the coarse-grained material due to the greater total grain boundary area to impede dislocation movement in fine grain material. The increase in hardness at locations of increasing numbers of grain boundaries corresponded to an increased yield stress in this region, as hardness measured the material’s resistance to plastic deformation. Furthermore, yield strength is inversely proportional to average grain size, as stated by the Hall–Petch relationship:

(2) $$\sigma_{y}=\sigma_{0}+k_{y}d^{-\frac{1}{2}},$$

where $\sigma_{y}$ is yield stress, $\sigma_{0}$ is a material constant for the starting stress for dislocation movement (or resistance of the lattice to dislocation movement), $k_{y}$ is strengthening coefficient (constant that is specific to each individual material), and d is average grain diameter [11,12].

The presence of strengthening precipitates such as γ’ should be considered when hardness is assessed, as, if an uneven distribution of these precipitates is present, hardness is affected. Areas where more strengthening precipitates are present have increased hardness. Figure 5a–c show strengthening precipitates were evenly distributed throughout the microstructure, but a variation in size of these precipitates was observed throughout the HAZ.

Results showed the same trend for all investigated parameters of a finer microstructure and higher hardness within and directly surrounding the bondline.

### 3.6. Porosity

**Figure 10:**
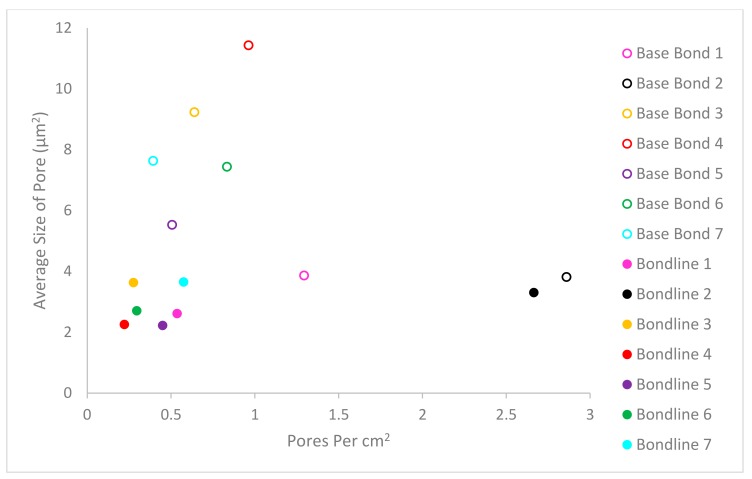
Graph showing pore size vs. pore number.

**Figure 11:**
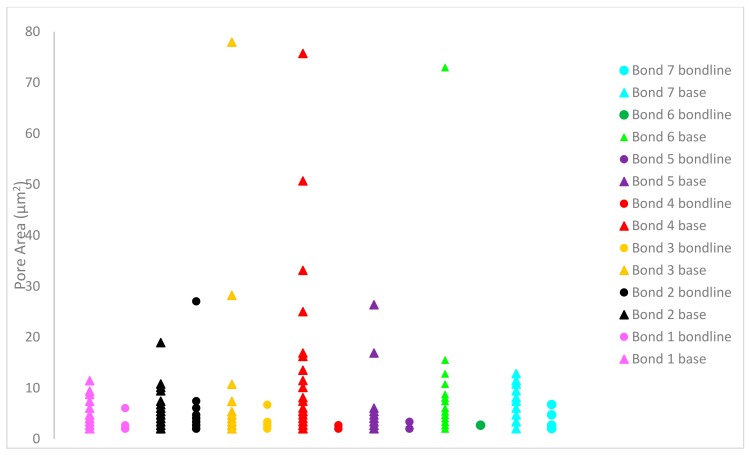
Pore-size distribution.

Analysis of the porosity results showed average bondline pore size to be smaller than the base material pore size. All specimens (except for bond 7) contained more pores per unit area in the base material than the bondline itself as observed in Figure 10. When the distribution of pore size was considered in Figure 11, there was also greater variation in pore sizes in the base material than within the bondline. Pores remaining in the base material after the PIB process were expected, as the as-received material contained pores throughout. The presence of pores post-bonding, indicated that the process did not alter the presence of the pores in the base material, but resulted in a relatively pore-free bondline, providing confidence that the inclusion of a powder interlayer results in a relatively pore-free structure. Considering the various bonding parameters, no clear trend existed between them and the resultant pore size or number.

## 4. Conclusions

Varying parameters such as temperature influences the driving-force degree for recrystallization to occur during PIB. At higher temperatures, there is more energy for recrystallization to occur, resulting in a change in the microstructure from the starting base material. Recrystallization in the bond region leads to more refined grain size than in the base material in the as-received condition. The finer microstructure at the bondline corresponded to increased hardness values in this area, as supported by the Hall–Petch relationship.

For the nickel-based superalloy investigated for this paper, the produced bondlines for PIB proved to be less porous than those in the surrounding base material, proving to be promising for the mechanical integrity of the bonded region.

## Figures and Tables

**Table 1:** Bonding parameters and resultant upset for each bond.

Bond Number	Time (min)	Force (kN)	Temperature (°C)	Deformation (%)
1	5–30	10–0.5	1100	14.16
2	10–30	10–0.5	1100	13.80
3	5	10	1100	3.13
4	5	10	1050	0.26
5	5	10	1100	3.00
6	10	5	1100	1.20
7	10	5	1050	0.36
8	10–30	5–0.5	1050	0.70
9	10	10	1050	1.80
10	10	10	1000	0.10
11	5–30	10–0.5	1100	26.60
12	10–30	10–0.5	1050	2.40

**Table 2:** EBDS grain-size data.

Grain Size Data from EBSD	
**Average gain size (µm)**	23
Minimum grain size (µm)	5.5
Maximum grain size (µm)	76
Number of measured grains	247
